# Wczesne Czynniki Ryzyka Rozwoju Nieswoistych Zapaleń Jelit w Populacji Pediatrycznej Województwa Kujawsko‒pomorskiego

**DOI:** 10.34763/devperiodmed.20182204.341350

**Published:** 2019-01-14

**Authors:** Aleksandra Dolińska, Zuzanna Wasielewska, Aneta Krogulska

**Affiliations:** 1 Katedra i Klinika Pediatrii, Alergologii i Gastroenterologii, Collegium Medicum w Bydgoszczy Uniwersytet Mikołaja Kopernika w Toruniu, Polska

**Keywords:** nieswoiste zapalenia jelit, wczesne czynniki ryzyka, dzieci, Inflammatory bowel disease, early risk factors, children

## Abstract

**Wstęp:**

*W ostatnich latach obserwuje się stałe zwiększanie częstości nowych zachorowań na nieswoiste zapalenia jelit u dzieci, stąd na całym świecie trwają badania mające na celu określenie prawdopodobnych czynników predysponujących do wystąpienia choroby*.

**Cel pracy:**

*Ocena występowania wczesnych czynników ryzyka u dzieci z nieswoistym zapaleniem jelit*.

**Metodyka:**

*Do grupy badanej zakwalifikowano 60 dzieci z nieswoistym zapaleniem jelit, w wieku 2-19 lat, będących pod opieką Kliniki Pediatrii, Alergologii i Gastroenterologii. Rozpoznanie choroby ustalono na podstawie obowiązujących kryteriów [ESPGHAN]. Wśród metod badanych zastosowano zwalidowany kwestionariusz ankiety własnej konstrukcji. Grupę kontrolną stanowiło 60 dzieci bez nieswoistego zapalenia jelit*.

**Wyniki:**

*Analiza drogi porodu w grupie badanej wykazała, że 14 (23%) dzieci zostało urodzonych drogą cięcia cesarskiego, a 46 (77%) siłami natury. U dzieci urodzonych drogą cięcia cesarskiego ryzyko wystąpienia choroby Leśniowskiego-Crohna było nieistotnie większe, natomiast u dzieci z wrzodziejącym zapaleniem jelita grubego - nieistotnie mniejsze w porównaniu z grupą kontrolną. Średni czas karmienia piersią w grupie badanej był nieistotnie krótszy w porównaniu z grupą kontrolną. Wcześniactwo i ekspozycja na dym nikotynowy w 1 roku życia zwiększały nieistotnie ryzyko wystąpienia wrzodziejącego zapalenia jelita grubego. Ciężki przebieg choroby dotyczył prawie połowy dzieci narażonych na antybiotykoterapię w 1rż., ale nie była to różnica istotna w porównaniu do dzieci o łagodniejszym przebiegu nieswoistego zapalenia jelit*.

**Wnioski:**

*Nie wykazano istotnych różnic w zakresie występowania wczesnych czynników ryzyka u dzieci z nieswoistym zapaleniem jelit w porównaniu do zdrowych dzieci. Wyłonienie czynników mogących mieć wpływ na rozwój choroby wymaga dalszych badań*.

## Abstract (en)

**Introduction:**

*In recent years there has been an increase in the incidence of new cases of inflammatory bowel disease in children, which is why research is underway to determine the likely predisposing factors for the disease*.

**Aim of the study:**

*To assess the prevalence of early risk factors in children with inflammatory bowel disease*.

**Methods:**

*The study included 60 children with inflammatory bowel disease, aged 2-19 years. Diagnosis was based on the current criteria [ESPGHAN]. A validated self-construction questionnaire was one of the methods used. The control group comprises 60 children without inflammatory bowel disease*.

**Results:**

*The analysis of the delivery route in the study group showed that 14 (23%) of the children were born via Caesarean section, and 46 (77%) by vaginal delivery. In the children born via C-section, the risk of Crohn’s disease was insignificantly higher than in the control group. The average breastfeeding time in the study group was insignificantly shorter compared to the control group. Prematurity and exposure to nicotine smoke in the first year of life negligibly increased the risk of ulcerative colitis. The severe course of the disease affected almost half of the children exposed to antibiotics in the 1st year, but it was not a significant difference in comparison to children with a milder course of the disease*.

**Conclusions:**

*There were no significant differences in the prevalence of early risk factors in children with inflammatory bowel disease compared to healthy children. The selection of factors that may affect the development of the disease requires further research*.

## Wstęp

Nieswoiste zapalenia jelit (NZJ) stanowią grupę schorzeń charakteryzujących się przewlekłym procesem zapalnym o złożonej i prawdopodobnie wieloczynnikowej etiologii. Dwiema najczęściej występującymi jednostkami chorobowymi w tej grupie są choroba Leśniowskiego - Crohna (ch. L-C) oraz wrzodziejące zapalenie jelita grubego (WZJG). Na NZJ wskazywać mogą objawy takie jak: nawracające bóle brzucha, biegunka (czasami z domieszką krwi lub śluzu), niedokrwistość, utrata masy ciała lub też niezwiązane z infekcją podwyższenie wykładników stanu zapalnego.

W ciągu ostatnich lat obserwuje się stałe zwiększanie częstości nowych zachorowań na NZJ [1]. Porównując lata 2007-2009 oraz 1998-2006 Jakobsen i wsp. wykazali wzrastającą częstość zachorowań na NZJ w populacji duńskiej [2]. Hope i wsp. badając dzieci irlandzkie odnotowali istotny wzrost zachorowań zarówno na ch. L-C, jak i WZJG w 2008 roku w stosunku do 2001 roku [3]. Porównując lata 2003-2008 z latami 1990-1995 Henderson i wsp. odnotowali znaczący wzrost zachorowań na NZJ w Szkocji [4]. Oprócz narastającego trendu zachorowalności na NZJ, niepokojącym zjawiskiem jest obniżanie się wieku wystąpienia pierwszych objawów choroby. Wykazano obniżenie mediany wieku zachorowania z 12,7 lat w latach 1990-1995 do 11,9 lat w latach 2003-2008 [4].

Wyniki przeprowadzonych dotychczas badań wskazują na udział w patogenezie NZJ nie tylko czynników genetycznych, ale również środowiskowych [5]. Uznaje się, że rozwój NZJ wynika z niekontrolowanej odpowiedzi zapalnej u osób genetycznie predysponowanych na dotychczas niezidentyfikowane czynniki środowiskowe, które oddziałują z mikrobiotą jelitową wpływając na układ immunologiczny i przewód pokarmowy [6]. Wydaje się, że narastającego trendu występowania NZJ nie można wytłumaczyć zmianami w genomie, bowiem okres czasu, w którym miałoby do nich dojść jest zbyt krótki. Szczególne zainteresowanie budzą więc czynniki środowiskowe, tym bardziej, że są one często modyfikowalne. Poznanie ich może zatem potencjalnie przyczynić się do wprowadzenia nowych metod profilaktyki bądź leczenia NZJ.

Ludzkie jelito stanowi przykład złożonego ekosystemu, w którym znajduje się bogata i zróżnicowana mikrobiota, która posiada kluczowy wpływ na prawidłowe funkcjonowanie przewodu pokarmowego człowieka. Na rozwój mikrobioty już od najwcześniejszego okresu życia ma wpływ wiele czynników, np. sposób karmienia dziecka, przebyte infekcje czy też antybiotykoterapia w pierwszych latach życia oraz droga porodu. Kolejnym ważnym elementem wpływającym na kształtowanie się mikrobioty przewodu pokarmowego jest rodzaj karmienia w pierwszym roku życia (karmienie naturalne vs karmienie sztuczne). Istotną rolę może odgrywać również fakt przebycia w pierwszych miesiącach życia antybiotykoterapii, co ma wpływ zarówno na bakterie chorobotwórcze, jak również mikrobiotę fizjologiczną.

Biorąc pod uwagę znaczenie wczesnego okresu życia dziecka na jego zdrowie w przyszłości, możliwy jest związek stanu noworodka po urodzeniu, sposobu odżywiania oraz innych czynników okołoporodowych z rozwojem NZJ. Kolejnym czynnikiem, o udokumentowanym znaczeniu, w rozwoju chorób cywilizacyjnych, w tym NZJ, jest palenie papierosów [7]. Na znaczenie czynników środowiskowych w patogenezie NZJ wskazuje również fakt, że dzieci imigrantów po przeprowadzeniu się do kraju o wyższej zachorowalności chorują częściej, niż rodzima populacja [8].

Celem badania jest ocena częstości występowania i związku wybranych czynników środowiskowych w okresie pre- i postnatalnym z rozwojem NZJ w grupie dzieci pozostających pod opieką Kliniki Pediatrii, Alergologii i Gastroenterologii w Bydgoszczy.

## Materiał i metody

Początkowo badaniem objęto 105 pacjentów z NZJ pozostających pod stałą opieką Kliniki Pediatrii, Alergologii i Gastroenterologii CM SU nr 1 w Bydgoszczy w okresie od 1 stycznia 2015 do 30 czerwca 2016 r. Grupę badaną stanowiło 66 dzieci z NZJ, z czego u 36 (54,55%) rozpoznano ch. L-C., u 24 (36,36%) WZJG, a u 6 (9,09%) NZJ nieokreślone. Z uwagi na niską liczebność grupy dzieci chorujących na postać nieokreśloną NZJ, pacjentów tych pominięto w dalszej analizie. Zatem ostatecznie do badanej grupy zaliczono 60 dzieci. Schemat przebiegu badania zamieszczono na Rycinie 1. NZJ rozpoznawano na podstawie obowiązujących kryteriów klinicznych, endoskopowych, histopatologicznych i radiologicznych zgodnie z zaleceniami ESPGHAN [9]. Wśród metod badawczych zastosowano zwalidowany kwestionariusz ankiety własnej konstrukcji. Grupę kontrolną stanowiło 60 zdrowych dzieci bez NZJ z Poradni Podstawowej Opieki Zdrowotnej w Bydgoszczy. Ankieta składała się z 13 pytań dotyczących wczesnych czynników ryzyka okresu okołoporodowego i niemowlęcego, takich jak: czas trwania i przebieg ciąży, droga porodu, masa urodzeniowa, narażenie na dym tytoniowy czy przebyte infekcje w 1 roku życia.

Zgodnie z klasyfikacją paryską badane dzieci podzielono na trzy grupy wiekowe: < 10 lat, 10-17 lat oraz ≥ 17 lat [10]. W analizie przebiegu klinicznego choroby za wyznacznik ciężkości przebiegu przyjęto konieczność stosowania leczenia biologicznego: przebieg ciężki – leczenie biologiczne, przebieg łagodny/umiarkowany – brak takiego leczenia.

Średni wiek rozpoznania ch. L.-C. wynosił 12,04 ± 3,42 lat (mediana 12 lat), a WZJG 10,60 ± 4,27 lat (mediana 11 lat) (p>0,05). U 15 (25,00%) dzieci z NZJ, chorobę rozpoznano w wieku poniżej 10 lat, u 5 (8,33%) w wieku 17 lat i powyżej. Wiek rozpoznania NZJ u pozostałych dzieci, tj. u 40 (66,67%) mieścił się między 10-17 lat. W badanej grupie u 22 (36,67%) dzieci stosowano leczenie biologiczne. Charakterystykę grupy badanej i kontrolnej przedstawiono w tabeli I.

Metoda statystyczna: W przypadku danych o charakterze ilościowym wyniki opisano przy użyciu następujących parametrów: liczba przypadków (N), wartość średnia (M), odchylenie standardowe (SD), minimum (Min), maksimum (Max) oraz mediana (Me). Dane o charakterze zmiennych jakościowych (kategoryzowanych) zostały opisane przez zestawienie względnej liczby przypadków (N) i ich procentowego udziału w badanej grupie. W celu wykrycia istnienia pomiędzy badanymi grupami ewentualnych różnic w wartościach poszczególnych czynników ilościowych zastosowano test *U* Manna-Whitneya, a dla rozkładów zmiennych kategoryzowanych – test chi-kwadrat. W celu wyodrębnienia czynników mających istotne znaczenie dla wystąpienia WZJG i ch. L-C zastosowano model regresji logistycznej (krokowej, postępującej) oraz iloraz szans wraz z podaniem przedziałów ufności. Iloraz szans dla zmiany jednostkowej zastosowanych parametrów oraz iloraz szans dla zmiany równej zakresowi analizowanych zmiennych został obliczony z 95% przedziałami ufności. We wszystkich porównaniach przyjęto poziom istotności statystycznej p<0.05. Analizę wyników badań przeprowadzono przy użyciu programu komputerowego STATISTICA 13.1 (Statsoft, Kraków, Polska).

## Wyniki

### Czas trwania i sposób rozwiązania ciąży

Analiza drogi porodu w grupie badanej wykazała, że 14 (23,33%) dzieci zostało urodzonych drogą cięcia cesarskiego, a 46 (76,67%) siłami natury. Wśród dzieci z rozpoznaną ch. L-C jedenaścioro (30,56%) urodziło się drogą cięcia cesarskiego, a 25 (69,44%) siłami natury, natomiast wśród dzieci z WZJG troje (12,50%) urodziło się drogą cięcia cesarskiego, a 21 (87,50%) siłami natury. W grupie kontrolnej siłami natury urodziło się 46 (76,67%) dzieci, a drogą cięcia cesarskiego – 14 (23,33%) badanych. Nie wykazano różnic w zakresie drogi porodu między dziećmi z NZJ a dziećmi z grupy kontrolnej (p>0,05), oraz między dziećmi z ch. L-C a dziećmi z WZJG (p>0,05). Średni czas trwania ciąży u matek dzieci z grupy badanej wynosił 39,27±1,73 i był porównywalny z grupą kontrolną (p>0,05). Z pierwszej ciąży urodziło się 20 (55,56%) dzieci z ch. L-C, 9 (37,50%) z WZJG oraz 33 (55,00%) dzieci z grupy kontrolnej. Porównując te trzy grupy wykazano, że dzieci z WZJG nieistotnie rzadziej pochodziły z pierwszej ciąży niż dzieci z ch. L-C oraz dzieci z grupy kontrolnej (p>0,05).

**Ryc. 1:**
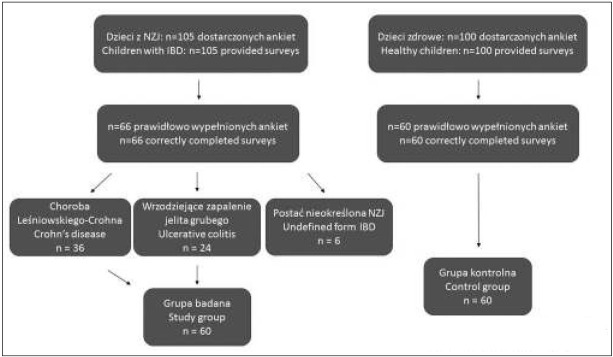
Schemat przebiegu badania Fig. 1. Study course diagram

**Table I:** Characteristics of the study and control groups at the time of data collection. Tabela I. Charakterystyka grupy badanej i kontrolnej w chwili zbierania danych.

Dane	Grupa badana *Study group* n=60 (100%)			Grupa kontrolna *Control group*	p
Data	Ch. L-C	WZJG	Razem	n=60 (100%)	
	*CD*	*CU*	*All*		
	n=36 (100%)	n=24 (100%)	n=60 (100%)		
Wiek w latach					
*Age in years*					
M ± SD	15,0 ± 3,08	14,3 ± 3,89	14,72±3,41	14,85 ± 2,92	0.231
*Me*	15,38	14,71	15.16	14,0	
min-max	4,75-18,5	6,08-19,25	4,75-19,25	6,58-18,71	

Płeć n (%)					
*Sex*					
Chłopcy			35 (58)	26 (43)	
*Boys*	21 (58)	14 (58)			0.144
Dziewczęta	15 (42)	10 (42)	25 (42)	34 (57)	
*Girls*					

Wiek w chwili rozpoznania					
w latach					
*Age at the time*					
*of diagnosis in years*					0.144
M ± SD					
Me	10.58 ± 4.24 11.00	11.42 ± 3.43 12.00	-	-	
min-max	3.00-17.00	2.00-17.00			

### Poronienia w wywiadzie u matek

U dziewięciu (15%) matek dzieci z NZJ występowały poronienia co najmniej 1 raz w życiu przed urodzeniem badanego dziecka, z czego 6 (16,67%) stanowiły matki dzieci z ch. L-C, a 3 (12,50%) matki dzieci z WZJG. Analogiczne dane w grupie kontrolnej dotyczyły matek 7 (11,67%) dzieci (w każdym przypadku p>0,05).

### Masa urodzeniowa

Troje (5%) dzieci z NZJ urodziło się z masą < 2500 g, z czego jedno choruje na ch. L-C (co stanowi 2,78% dzieci z ch. L-C), a 2 dzieci na WZJG (co stanowi 8,33% dzieci z WZJG). W grupie kontrolnej 5 (8,33%) dzieci urodziło się z niską urodzeniową masą ciała. Nie wykazano istotnych różnic w zakresie średniej masy urodzeniowej między dziećmi z NZJ a grupą kontrolną, jak również między dziećmi z ch. L-C a WZJG (p>0,05)

### Punktacja w skali Apgar

Średnia ocena w skali Apgar dzieci z grupy badanej wynosiła 9,15 ±1,16 i była porównywalna z oceną dzieci z grupy kontrolnej. Większość pacjentów z grupy badanej, jak i kontrolnej – odpowiednio 55 (91,67%) oraz 56 (93,33%) - urodziło się w stanie dobrym, z punktacją 8-10 pkt. w skali Apgar. Punktacja ta dotyczyła 29(58,00%) dzieci z ch. L-C oraz 21 (42,00%) dzieci w WZJG.

### Karmienie piersią

Średni czas karmienia piersią (wyłącznie lub w sposób mieszany) w grupie badanej był nieistotnie krótszy w porównaniu z grupą kontrolną (p>0,05). Wyłącznie karmionych piersią przez 1-6 miesięcy było 24 (40%) dzieci, z czego u 13 (54,17%) rozpoznano ch. L-C (co stanowi 36,10% dzieci z tym rozpoznaniem), natomiast u 11 (45,83%) dzieci - WZJG (co stanowi 45,83% dzieci z tym rozpoznaniem). W grupie kontrolnej wyłączne karmienie piersią dotyczyło 25 (41,70%) pacjentów.

### Antybiotykoterapia w 1. roku życia dziecka

Antybiotyk w 1. roku życia stosowano u 22 (36,67%) dzieci z NZJ oraz u 24 (40%) dzieci w grupie kontrolnej (p>0,05). Nieistotnie częściej antybiotykoterapia dotyczyła dzieci z ch. L-C, tj. 15 (41,67%), w porównaniu z pacjentami z WZJG, tj. 7 (29,17%), (p>0,05).

### Hospitalizacje w 1. roku życia dziecka

W pierwszym roku życia 16 (26,67%) dzieci z NZJ było choć raz hospitalizowanych. W grupie kontrolnej dotyczyło to 20 (33,33%) dzieci, (p>0,05). Wśród dzieci, które były hospitalizowane w 1 roku życia 10 (62,50%) stanowiły dzieci z ch. L-C (co stanowi 27,78% dzieci z tym rozpoznaniem) oraz 6 (37,50%) z WZJG (co stanowi 25% badanych z tym rozpoznaniem).

### Ekspozycja na dym tytoniowy w okresie ciąży

Matki 6 (10%) dzieci z NZJ paliły papierosy w czasie trwania ciąży, z czego u 2 dzieci rozpoznano ch. L-C, a u 4 WZJG, natomiast w grupie kontrolnej dotyczyło to 4 (6,67%) matek (p>0,05).

### Ekspozycja na dym tytoniowy w okresie niemowlęcym

W badanej grupie dzieci, na ekspozycję na dym tytoniowy (rozumianą jako palenie papierosów przez matkę lub innego domownika) w 1 roku życia narażono 26 (43,33%) dzieci z NZJ, natomiast w grupie kontrolnej - 24 (40%) dzieci, (p>0,05).

W grupie badanej eksponowanych na działanie dymu tytoniowego w okresie niemowlęcym było 16 (44,44%) dzieci z ch. L-C, oraz 10 (41,67%) dzieci z WZJG.

Powyższe dane przedstawiono w tabeli II.

**Tabela II:** Wczesne czynniki ryzyka w grupie badanej i kontrolnej. *Table II. Early risk factors in the study and control group*.

Badana zmienna Risk factor	Dzieci z ch. L-C CD n=36 (100%)	Dzieci z WZJG CU n=24(100%)	Dzieci z NZJ razem Total IBD n=60(100%)	Grupa kontrolna Control group n=60(100%)	p*	p#
Wcześniactwo, n (%)	1(3)	6(25)	7(12)	8(13)	0,99	0,01
*Prematurity, n (%)*						
Poronienia, n (%)	6(17)	3(13)	9(15)	7(12)	0,79	0,73
*Miscarriage, n (%)*						
Urodzenia z pierwszej ciąży, n (%)	20 (56)	9 (38)	29 (48)	33 (55)	0,58	0,20
*Births from 1st pregnancy, n (%)*						
Rodzaj porodu, n (%)						
*Type of delivery, n (%)*						
Siłami natury	25 (69)	21 (88)	46 (77)	46 (77)	1	
*Natural* Cięcie cesarskie *Caesarean* *section*	11 (31)	3(13)	14 (23)	14 (23)	1	0,13
Hbd, M ± SD	39,33 ± 1,64	38,96 ± 1,79	39,27 ± 1,73	39,23 ± 2,52	0,93	0,74
Urodzeniowa masa ciała, g, M ± SD						
*Birth weight, g, M± SD*	3355 ± 505,78	3513,33 ± 476,63	3410 ± 521,5	3386 ± 688,4	0,83	0,16
Punktacja w skali Apgar, M ± SD						
*Apgar scores, M±SD*	9,17 ± 1,11	9,08 ± 1,25	9,15 ± 1,16	9,27 ± 1,40	0,62	0,75
Długość karmienia piersią, m-ce, M ± SD						
*Duration of breastfeeding, months, M±SD*	7,36 ± 9,02	7,71 ± 8,04	7,70 ± 8,30	8,82 ± 9,68	0,50	0,96
Ekspozycja na dym tytoniowy, n (%)						
*exposure to tobacco smoke, n (%)*						
*w* ciąży						
*during pregnancy*	2(6)	4(17)	6(10)	4(7)	0,74	0,20
*w* domu w 1 rż						
*at home up to 12^th^ mth*	16 (44)	10 (42)	27 (45)	24 (40)	0,85	0,52
Antybiotykoterapia w 1rż, n (%)	15 (42)	7(29)	22 (37)	24 (40)	0,85	0,41
*Antibiotic therapy in 1 year, n (%)*						
Hospitalizacje w 1 rż, n (%)						
*Hospitalization in 1 year, n (%)*	10 (28)	6(25)	16 (27)	20 (33)	0,55	0,53

* porównanie grupy badanej z grupą kontrolną/comporison *of the study group with the control group #*porównanie ch. L-C z WZJG*/comparison of the CD with CU*

### Ryzyko wystąpienia NZJ w zależności od wybranych czynników środowiskowych

Celem oceny znaczenia wybranych czynników środowiskowych w rozwoju NZJ przeprowadzono wieloczynnikową analizę regresji logistycznej. Nie wykazano występowania istotnego związku NZJ z wcześniactwem, kolejnością urodzenia, rodzajem porodu i antybiotykoterapią, hospitalizacją w 1 roku życia oraz ekspozycją na dym tytoniowy (tab. III). Ryzyko wystąpienia ch. L-C było nieistotnie większe u dzieci urodzonych drogą cięcia cesarskiego (OR=1,7; 0,71-4,21; p>0,05), natomiast u dzieci z WZJG - nieistotnie mniejsze (OR=0,4; 0,11–1,48; p>0,05). Wcześniactwo i ekspozycja na dym nikotynowy w 1 roku życia zwiększały nieistotnie ryzyko wystąpienia WZJG (OR = 2,17; 0,66-7,91; p>0,05) (OR=2,8; 0,64-12,26; p>0,05).

**Table III:** Risk assessment of CD and CU depending on selected environmental factors. Tabela III. Ocena ryzyka wystąpienia ch. L-C i WZJG w zależności od wybranych czynników środowiskowych.

	Dzieci z NZJ		
	*Kids with IBD*		
Badana zmienna	n=60 (100%)		
*Risk factor*			
	OR [CI 95%] dla ch. L-C	OR [CI 95%] dla WZJG	
	*OR [CI 95%] for CD*	p *OR [CI 95%] for CU*	p
Wcześniactwo, n (%)	0,18 [0,02 – 1,55]	0,15 2,17 [0,66- 7,91]	0,33
*Prematurity, n (%)*			
Poronienia, n (%)			
*Miscarriage, n (%)*	1,51 [0,47-4,92]	0,54 1,08 [0,25-4,58]	0,59
Urodzenia z pierwszej ciąży, n (%)			
*Births from 1st pregnancy, n (%)*	1,25 [0,57 – 2,74]	0,58 0,58 [0,19 – 1,22]	0,12
Rodzaj porodu, n (%)			
*Type of delivery, n (%)*			
Siłami natury			
*Natural*	0,58 [0,24 – 1,40]	0,22 2,46 [0,68 – 8,98]	0,17
Cięcie cesarskie			
*Caesarean section*	1,73 [ 0,71 – 4,21]	0,22 0,41 [0,11 – 1,48]	0,17
Ekspozycja na dym tytoniowy, n (%)			
*Exposure to tobacco smoke, n (%)*			
w ciąży			
*during pregnancy*	0,82 [0,14-4,74]	0,60 2,80 [0,64-12,26]	0,16
w domu w 1rż			
*at home during 1^st^ year*	1,18 [0,53 – 2,59]	0,69 1,00 [0,40 – 2,48]	1,00
Antybiotykoterapia w 1rż, n (%)			
*Antibiotic therapy in 1^th^ year, n (%)*	1,07 [0,46-2,48]	0,86 0,62 [0,22 - 1,71]	0,42
Hospitalizacje w 1rż, n (%)			
*Hospitalization in 1^th^ year, n (%)*	0,86 [0,36 – 2,03]	0,73 0,73 [0,26 – 2,03]	0,55

### Analiza związku między wybranymi czynnikami środowiskowymi a płcią, czasem rozwoju i ciężkością NZJ

Choć wykazano różnice w częstości antybiotykoterapii, hospitalizacji w 1rż, okresie karmienia piersią w zależności od płci dzieci z NZJ, oraz stwierdzono, że NZJ o wczesnym początku częściej dotyczył dzieci poddanych ekspozycji na dym tytoniowy w ciąży niż NZJ, który rozwinął się u dzieci po 10rż, to różnice te były nieistotne statystycznie. Ciężki przebieg choroby dotyczył prawie połowy dzieci narażonych na antybiotykoterapię w 1rż., ale nie była to różnica istotna w porównaniu do dzieci o łagodniejszym przebiegu NZJ (tab. IV).

**Tabela IV:** Analiza wybranych czynników środowiskowych w zależności od płci, wieku w momencie rozpoznania oraz ciężkości NZJ. *Table IV. Analysis of selected environmental factors depending on sex, age at the time of diagnosis and the severity of IBD*.

Badana Risk zmienna factor	Płeć Sex		P	Wiek przy rozpoznaniu [lata] *Age at the time of diagnosis [years]*			P	Leczenie biologiczne *Biological treatment*		P
	Chłopcy *Boys* n=35 (100%)	Dziewczynki *Girls* n=25 (100%)		< 10 n=15 (100%)	10-17 n=44 (100%)	> 17 n=l (100%)		Tak *Yes* n=22 (100%)	Nie *No* n=38 (100%)	
Wcześniactwo, n (%)	4(11)		0,93			0	0,24	0		0,27
*Prematurity, n (%)*		3(12)		1(7)	6(14)				7(18)	
Poronienia, n (%)										
*Miscarriages, n (%)*	3(9)	6(24)	0,89	0	9 (20)	0	0,28	5(23)	4(11)	0,15
Urodzenia z pierwszej ciąży, n (%)										
*Births from 1st pregnancy, n (%)*	17 (49)	12 (48)	0,84	8 (53)	21(48)	0	0,38	11 (50)	18 (47)	0,53
Rodzaj porodu, n (%)										
*Type of delivery, n (%)*										
Siłami natury	26 (74)	20 (80)		11(73)	34 (77)	1 (100)		14 (64)	32 (84)	
*Natural*										
Cięcie cesarskie	9(26)	5(20)	0,35	4(27)	10 (23)	0	0,43	8(36)	6(16)	0,11
*Caesarean section*										
Hbd, M ± SD	39,09 ± 1,70	39,32 ± 1,63	0,80	39,40 ± 1,71	39,09 ± 1,66	40,00	0,54	39,32 ± 1,66	39,11 ± 1,20	0,89
Urodzeniowa masa ciała,										
g, M ± SD	3339,14 ± 520,17	3529,2 ± 502,14	0,50	3414,0 ± 513,83	3417,95 ± 507,13	3500	0,93	3429,55 ± 504,67	3411,84 ± 516,63	0,62
*Birth weight, g, M± SD*										
Punktacja w skali Apgar, M ± SD	9,09 ± 1,14	9,20 ± 1,09	0,22	9,60 ± 1,08	8,95 ± 1,14	10	0,06	9,09 ± 1,16	9,16 ± 1,07	0,74
*Apgar scores, M± SD*										
Długość karmienia piersią,										
m-ce, M ± SD	8,71 ± 8,61	5,80 ± 8,98	0,08	9,00 ± 8,68	7,16 ± 8,85	0	0,23	9,45 ± 8,78	6,37 ± 8,78	0,27
*Duration of breastfeeding*,										
*months, M± SD*										
Ekspozycja na dym										
tytoniowy, n (%)										
*Exposure to tobacco*										
*smoke, n (%)*	4(11)		0,86			1 (100)	0,63			0,53
w ciąży		2(8)		2(13)	3(7)			1(5)	5(13)	
*during pregnancy*										
w domu w 1rż	17 (49)	10 (40)	0,57	6 (40)	20 (45)	1 (100)	0,80	10 (45)	17 (45)	0,37
*at home in 1^st^ year*										
Antybiotykoterapia w 1rż, n (%)	10 (29)	12 (48)	0,09	6 (40)	16 (36)	0	0,69	10 (45)	12 (32)	0,28
*Antibiotic therapy in 1^st^ year, n (%)*										

## Dyskusja

Mimo badań prowadzonych od lat patogeneza NZJ nadal nie jest znana. Wśród potencjalnych czynników sprawczych bierze się pod uwagę predyspozycję gene-tyczną, zmiany mikrobioty jelitowej, defekty odporności nabytej i wrodzonej oraz inne czynniki środowiskowe [11]. Wyłonienie czynników ryzyka choroby mogłoby przyczynić się do określenia nowych metod terapii i prewencji NZJ.

W badaniu własnym nie wykazano istotnych różnic w zakresie występowania wybranych czynników środowiskowych, takich jak: czynniki okołoporodowe, antybiotykoterapia i hospitalizacje w 1 roku życia oraz ekspozycja na dym tytoniowy u dzieci z NZJ w porównaniu do zdrowych dzieci.

Dysbioza w zakresie mikrobioty przewodu pokarmowego bywa związana z wieloma schorzeniami, w tym również z NZJ. Charakteryzuje ją zmniejszona różnorodność tych bakterii przewodu pokarmowego, które wykazują działanie przeciwzapalne – np. Faecalibacterium oraz zwiększona ilość Enterobacteriaceae (np. E. coli) [12]. Znaczącą rolę mikrobioty w rozwoju NZJ oraz ich późniejszych zaostrzeń może pośrednio potwierdzać skuteczność nowej metody leczenia NZJ, tj. przeszczep bakterii jelitowych (Fecal Microbiota Transplantation), której celem jest nie tylko skorygowanie dysbiozy ale również przywrócenie prawidłowego współdziałania systemu immunologicznego oraz mikrobioty [13].

Zaburzenia w składzie mikrobioty przewodu pokarmowego mogą pojawić się już w najwcześniejszych latach życia. Wpływ na nią ma m.in. droga porodu, a co za tym idzie naturalna kolonizacja dziecka mikrobiotą matki podczas przejścia przez kanał rodny. Stąd też powstała hipoteza, że urodzenie metodą cięcia cesarskiego może prowadzić do dysbiozy, zwiększając ryzyko zachorowania na NZJ. Sevelsted i wsp. stwierdzili istotną statystycznie zależność pomiędzy urodzeniem dziecka metodą cięcia cesarskiego a wzrostem ryzyka późniejszego zachorowania na NZJ, astmę oraz ogólnoustrojowe choroby tkanki łącznej [14]. Również Bager i wsp. wykazali istotne statystycznie zwiększenie ryzyka zachorowania na NZJ u dzieci urodzonych drogą cięcia cesarskiego [15]. Malmborg i wsp. dowiedli, że narodziny przez cesarskie cięcie jest związane z nieznacznie zwiększonym ryzykiem wystąpienia ch. L-C u chłopców, a w przypadku cięcia elektywnego również u dziewcząt [16]. Z kolei Li i wsp. wykazali istotny związek pomiędzy porodem metodą cięcia cesarskiego a późniejszym zachorowaniem dziecka na ch. L-C, ale nie WZJG [17]. Badania ostatnich lat nie potwierdzają istotnych różnic w częstości zachorowań na NZJ pomiędzy dziećmi urodzonymi drogami natury oraz metodą cięcia cesarskiego [18, 19].

Według najnowszych danych pochodzących z 150 krajów na całym świecie, obecnie 18,6% wszystkich porodów odbywa się drogą cięcia cesarskiego, a w Europie wskaźnik ten sięga 25% [20]. W badaniu własnym wykazano, że wśród dzieci z ch. L-C, odsetek dzieci urodzonych przez cesarskie cięcie był wyższy (31%), niż w przypadku pacjentów z WZJG (13%). Jest to również wartość wyższa niż przeciętny odsetek cięć cesarskich wykonywanych w populacji ogólnej, co może wskazywać na potencjalny związek pomiędzy drogą porodu dziecka a późniejszym zachorowaniem na ch. L-C, choć porównanie częstości porodów drogą cięcia cesarskiego w grupie NZJ z dziećmi zdrowymi nie wykazało różnic.

Kolejnym istotnym czynnikiem ryzyka NZJ i innych chorób cywilizacyjnych jest stosowanie nieprawidłowej diety. Amre i wsp. wykazali zależność pomiędzy stosowaniem diety niezbilansowanej pod względem zawartości kwasów tłuszczowych, warzyw oraz owoców, a zwiększonym ryzykiem zachorowania na ch. L-C [21]. Nie wiadomo czy istnieje związek pomiędzy sposobem karmienia dziecka w okresie noworodkowym i niemowlęcym a późniejszym zachorowaniem na NZJ. Protekcyjna rola karmienia piersią została wielokrotnie udowodniona wobec podatności dziecka na infekcje [22, 23] czy rozwoju otyłości [24]. Na podstawie badań przeprowadzonych w populacji duńskiej Hansen i wsp. wykazali, że u dzieci karmionych piersią dłużej niż 6 miesięcy ryzyko rozwoju NZJ w kolejnych latach życia jest mniejsze [25]. Wyniki badań własnych nie potwierdziły takiej zależności. Odsetek dzieci, które w ogóle nie były karmione piersią był niski (13,64%), a liczba dzieci z NZJ, które były karmione pokarmem matki krócej niż 6 miesięcy oraz dłużej niż 6 miesięcy była identyczna (42,4%).

Zmiany równowagi mikrobioty jelitowej mogą być powodowane również poprzez stosowanie antybiotyków. Ponadto wiadomo, że leki te modulują odpowiedź immunologiczną, a tym samym mogą wpływać na rozwój NZJ. Wykazano związek pomiędzy antybiotykoterapią w wieku niemowlęcym i wczesnym dzieciństwie, a późniejszą zwiększoną predyspozycją do zachorowania na NZJ, zwłaszcza ch. L-C [26,27,28]. Na podstawie wyników badań własnych nie wykazano różnic w częstości stosowania antybiotyków w pierwszym roku życia między dziećmi z NZJ, a dziećmi zdrowymi. W przeprowadzonym badaniu nie wykazano również związku pomiędzy hospitalizacją w 1 roku życia a występowaniem NZJ.

Kolejnym ważnym czynnikiem rozwoju chorób cywilizacyjnych, w tym NZJ, jest narażenie na działanie dymu tytoniowego. Udowodniono, iż palacze z ch. L-C mają cięższy przebieg choroby niż osoby niepalące, a zaprzestanie palenia może złagodzić ten proces [29]. W badaniu własnym wykazano, że papierosy w czasie ciąży paliły matki 10% dzieci z NZJ, w większości dotyczyło to dzieci z WZJG. Analiza badań własnych nie potwierdziła związku między biernym narażeniem na działanie dymu tytoniowego w 1 roku życia (palenie papierosów przez matkę lub domowników) a rozwojem NZJ u badanych dzieci.

Jednym z celów naszego badania była próba oceny związku czynników okołoporodowych takich jak – czas trwania ciąży, urodzeniowa masa ciała oraz punktacja w skali Apgar z rozwojem NZJ w badanej populacji dzieci. Na podstawie przeprowadzonych badań nie wykazaliśmy jednak istotnych różnic w tym zakresie między dziećmi z NZJ a dziećmi zdrowymi.

Jak nam wiadomo, badanie własne jest pierwszym badaniem podejmującym próbę oceny związku wybranych czynników środowiskowych z rozwojem NZJ w populacji polskich dzieci. Słabym punktem badania jest mała liczebność grupy badanej, z czego może wynikać brak istotnych zależności statystycznych. Przeprowadzone badanie traktujemy jako badanie wstępne wymagające kontynuacji.

## Wnioski

Nie wykazano istotnych różnic w zakresie występowania wczesnych czynników ryzyka u badanych dzieci z NZJ w porównaniu do zdrowych dzieci. Wyłonienie czynników mogących mieć wpływ na rozwój NZJ wymaga dalszych badań.
